# Factors Influencing the Use of Pedestrian Bridges in North of Iran

**DOI:** 10.30476/BEAT.2022.92068.1297

**Published:** 2022-01

**Authors:** Zahra Mohtasham-Amiri, Iraj Barge-Gol, Leila Kouchakinejad-Eramsadati, Payam Abedian, Helya Jafari-Shakib

**Affiliations:** 1 *Guilan Road Trauma Research Center, Guilan University of Medical Sciences, Rasht, Iran *; 2 *Preventive and Social Medicine Department, Guilan University of Medical Sciences, Rasht, Iran*; 3 *Department of Civil Engineering, Guilan University, Rasht, Iran*

**Keywords:** Pedestrian bridges, Road traffic injuries, Behaviors, Guilan, Iran

## Abstract

**Objective:**

To find out factors that influence the use/non-use of pedestrian bridges in Northern Iran.

**Methods:**

In this observational study, 4 pedestrian bridges at four different places in Rasht, North of Iran was studied. In addition to demographic data, pedestrians were interviewed for reasons of use/not use the pedestrian bridge. The data analyzed with SPSS software version 18 by non-parametric tests such as Chi Square.

**Results:**

From all 499 participants, more than one-third of pedestrians had not used pedestrian bridges. The most reasons for bridges use among respondents were feeling of safety and security (79.2%) and obey the rules (53.6%). The reasons for the non-use of bridges were time saving (63.7%), laziness (48.7%) and inappropriate of the bridge (34.2%). There were no significant differences in age, sex, education level, and road accidents’ history with use of bridge but there was a significant difference between the bridge usage with having a driver’s license, rash-hour time, and the presence of a mechanical elevator (*p*<0.001).

**Conclusion:**

The results of this study show that in order to increase the pedestrian bridges use, it is necessary to pay more attention to make standard facilities such as installing escalators or elevators of these bridges.

## Introduction

Road traffic injuries (RTIs) result in the deaths of approximately 1.3 million people around the world each year and leave between 20 to 50 million people with non-fatal injuries. More than half of all road traffic deaths and injuries involve vulnerable road users such as pedestrians, cyclists and motorcyclists and their passengers [1]. Road accidents are projected to be the 4^th^ leading cause of global disease burden in the year 2030 [2, 3].

Road accidents in North Africa and the Middle East are one of the major public health problems and the leading cause of death [4]. Iran has one of the highest rate of road traffic injuries in the world with 35.6 (UI: 29.64–43.44) deaths per 100,000 and 1,436 life years affected due to disability from road crash injuries per 100,000 people [4, 5]. Unfortunately, 75% of road crash fatalities and injuries occur in the economically productive age groups (15-64 years.). According to road users, about 35% of RTIs in Iran are pedestrians with high fatality and severe injuries [5, 6]. This rate is higher than the global rate as 22% of all deaths [7].

Studies conducted in Iran show a high rate of mortalities and injuries related to RTIs among pedestrians [8-11]; therefore, pedestrian safety is one of the goals of the health and transportation system in Iran.

The pedestrian movement gradually became problematic with increasing the population, the creation of mega cities and metropolises and simultaneously the increasing number of motorized vehicles in recent decades. One of the best solutions is using pedestrian bridges for the safe pedestrians’ transportation. Pedestrian bridges are structures made for allowing pedestrians to cross a street/road/highway without being exposed to any risks of car vehicles. Indeed a pedestrian bridge removes pedestrians from vehicle roadway [12]. Unfortunately previous studies illustrated that most of pedestrians do not use pedestrian bridges properly for different reasons [12-21]. This both increases the risk of collisions for pedestrians and disrupts the flow of street traffic. Factors affecting the use of pedestrian bridges have been investigated in recent studies and the main factors for their usage were perceived safety and security, time and distance savings [20-22]. The objective of this study was to explore the factors associated of use or non use of pedestrian bridges in Rasht metropolitan which is the biggest city of Guilan,North of Iran with a very high population density and high rate of RTIs [23].

## Materials and Methods

This cross-sectional survey was conducted on four pedestrian bridges out of 11 bridges randomly in Rasht in the second half of March 2018. Half of them had both escalators and elevators. Pedestrians were selected non-randomly from the pedestrian bridge to a distance of 50 meters from it, and pedestrians were interviewed in a structured manner. The study was conducted during the weekdays from 7 am to 10 pm, the period of maximum activity of passers-by. High traffic volumes at all sites were noted by the survey team observational. The interview was done by the research team and the interviewees were divided into groups of pedestrian bridge users and those who did not use the bridge and crossed the street illegally. The pedestrian who crossed the roads outside 50 meters from the pedestrian bridge were recorded as not using the footbridge. It was not possible to observe all pedestrian crossings during the survey period. The research continued until the appropriate sample size. Participation in the study was not mandatory and individuals entered the study voluntarily. Finally, 499 pedestrians participated in this study non-randomly . Disabled people and pregnant women were excluded from the study.

In the interview, in addition to the age, gender, education level, having a driver’s license, and a history of road traffic accidents, perceptions of bridges as crossing facilities were asked by a semi-stractured questionaire. At the end of the questionnaire, there was an open question about other reasons for using or not using the bridge. The data were analyzed with SPSS software version 18 by non-parametric tests such as Chi Square and logistic regression model.

## Results

Of all participants, nearly two-thirds of pedestrians were men (62.3%), that their mean age was 34.8±12.2 years (from 9 to 76 years). The majority of pedestrians were among the 20-30 age group (33.7%), followed by the 31-40 age group (27.7%). The proportion of pedestrians aged under 18 was low (5%). Most of them had high school or academic education, respectively (43.7%, 26.3%) and 16.4% had history of road traafic injuries. More than one-third of pedestrians did not use pedestrian bridges (Table 1).

**Table 1:** Pedestrian characteristics and behaviors (n=499).

**Variables**		**n**	**%**
Gender	Male	311	62.3
	Female	188	37.7
Age group (yrs)	<20	44	8.8
	20-30	187	33.7
	31-40	138	27.6
	41-50	93	18.6
	>50	56	11.2
Education	Illitrate or <5 grade	62	12.4
	5-9	88	17.6
	9-12	218	43.7
	Academic	131	26.3
Self-history of road traffic injuries	Yes	136	27.3
	No	363	72.7
Family history of road traffic injuries	Yes	139	27.9
	No	360	72.1
Having driving license	Yes	291	58.3
	No	208	41.7
Usage of Pedestrian bridge	Yes	312	62.5
	No	187	37.5

Most purposes for road usage among respondents were going and returning home from work (27.6%) but more than 25% of pedestrians had no definite purpose for road using. Eighty-two individuals (16.4%) were with other family members or friends on the road and others had traveled alone.

Regarding the question “Who is to blame in the event of a pedestrian accident under the pedestrian bridge? “the majority of respondents believed the motor vehicles drivers and then pedestrians as 54.3% and 37.3%, respectively. Others had believed that it depended on police decisions.

The most reasons for bridge usage among respodents were feeling of safety and security (79.2%) and obey the rules (53.6%). The reasons for non-usage of bridges were time-saving (63.7%), laziness (48.7%), the inadequacy of the bridge (non-availability of escalator or elevator, the high number of steps, not having a roof, non-provision of lighting during the evening and night hours) (34.2%), slipping of stairs (28%), and Steep of the bridges (22%).

There was no significant differences in the age, sex, education level, and history of road accidents with using of the pedestrian bridge but there were a significant differences between using the bridge with having a driver’s license, rash-hour time and the presence of a mechanical elevator on the bridge (Table 2). There was no statistically significant difference between the mean and standard deviation of age in the two groups of users and non-users of the pedestrian bridge ( 34.6±12.7 vs. 35.1±11.5, *p*=0.65)

**Table 2:** Effective factors on usage of pedestrian bridges

**Variables**		**Usage ** **N (%)**	**Non- usage** **N (%)**	**Ρ-value**
Gender	Male	189 (60.8)	122 (39.2)	0.34
	Female	123 (65.4)	65 (34.6)	
Age group ( yrs)	<20	31 (70.5)	13 (29.5)	0.55
	20-30	108 (64.3)	60 (35.7)	
	31-50	138 (59.7)	93 (41.3)	
	>50	35 (62.5)	21 (37.5)	
Education	Illitrate or <5 grade	39 (62.9)	23 (37.1)	0.09
	5-9 grade	55 (62.5)	33 (37.5)	
	9-12 grade	125 (57.3)	93 (42.7)	
	>12 grade	93 (71)	38 (29)	
Self-history of road traffic injuries	Yes	85 (62.5)	51 (37.5)	0.53
	No	227 (62.5)	136 (37.5)	
Family history of road traffic injuries	Yes	86 (61.9)	53 (39.1)	0.91
	No	226 (62.8)	134 (37.2)	
Having driving license	Yes	171 (58.8)	120 (41.2)	0.04
	No	141 (67.8)	62 (32.2)	
Accompany person	Yes	54 (65.1)	120 (34.1)	0.53
	No	258 (62)	62 (38)	
Time- period	7:00-9:00	39 (73.6)	14 (26.4)	0.000
	9:01-11:00	65 (66.3)	33 (33.7)	
	11:01-12:30	53 (57.6)	39 (42.4)	
	12:31-14:00	30 (40)	45 (60)	
	14:01-16:00	43 (68.3)	20 (31.7)	
	16.01-18:00	29 (59.2)	20 (40.8)	
	18:00-20:00	53 (76.8)	16 (23.2)	
Escalator or elevator in Pedesterian bridge	Yes	143 (67.5)	69 (32.5)	0.03
	No	169 (58.9)	118 (41.1)	

After logistic regression model education, age group, existence of fences under the pedestrian bridge and escalator in pedestrian bridge were the effecting factors on usage of pedestrian bridges (*p*<0.05) and time-period (*p*<0.1) (Table 3).

**Table 3:** Final effective factors on usage of pedestrian bridges by logistic regression model

		**B**	**S.E.**	**P-value**
Step 1^a^	Gender: Male (Base)	-0.412	0.293	0.160
	Education: (Illiterate or <5 grade)base			0.006
	5-9 grade	-0.742	0.567	0.190
	9-12 grade	-1.046	0.465	0.024
	>12 grade	-1.140	0.334	0.001
	Accompany person: (no) base	-0.499	0.328	0.128
	Self-history of RTAs: (no) base	-0.157	0.289	0.586
	Family history of RTAs: (no) base	-0.233	0.284	0.413
	Having driving license: (no) base	0.154	0.329	0.638
	Age group: (<20 yrs) base			0.002
	20-30	2.223	0.681	0.001
	31-50	0.519	0.520	0.318
	>50	0.080	0.465	0.863
	Time- period: (7:00-9:00)base			0.585
	9:01-11:00	-0.620	0.653	0.342
	11:01-12:30	-0.611	0.385	0.113
	12:31-14:00	-0.559	0.410	0.173
	14:01-16:00	-0.713	0.518	0.169
	16.01-18:00	-1.050	0.598	0.079
	18:00-20:00	-0.850	0.479	0.076
	Existence of fences under the Pedestrian bridge: (No) base	4.490	0.485	0.000
	Escalator in Pedestrian bridge: (No) base	2.377	0.476	0.000
	Constant	-1.748	0.907	0.054

## Discussion

Pedestrians are one of the most high-risk groups in road traffic accidents in the world and in Iran [5-11]. Therefore, the facilities to cross the road, like the pedestrian bridge and underpasses are very important to lower crashes and mortality rates in this high- risk group. Certainly, the pedestrian bridge should be built in a suitable place with standard and complete safety conditions, therefore, pedestrians think that it is time-saving to use the bridge and they feel safe, comfortable and enjoy their pass. Therefore, it is very costly and requires extensive time to implement.

The effectiveness of a pedestrian bridge depends on how much it is used. However, previous studies show low use of pedestrian bridges by pedestrians in different geographic areas [12-21].

The largest pedestrian age group interviewed belonged to the 20-40 years old group totaling 63.3% of the respondents surveyed.

In this study, most of pedestrians used pedestrian bridges (62.5%). It is in concordance with previous studies in Vietnam (between 35.9% and 96.5%) [22], Turkey (from 6 to 63%) [18, 20], Colombia (65%) [17] and higher than studies in Mexico (50.5 %) [21], Malaysia (11.97%) [15], and Indonesia (13%) [16]. The use of pedestrian bridges is a complex behavioral phenomenon, involving many factors. Some factors depend on pedestrians and many depend on road and bridge situations. The main factor for the implementation of the bridge is decreasing RTIs in road users; therefore, it should be designed in a way that focuses more on human needs as a priority rather than the other two elements of the transportation system: road and vehicle. The bridge structure should be in such a way that the pedestrians feel comfortable, secure, and safe; they should enjoy their pass and find it time-saving.

There was not any difference between gender and the bridges use, while some studies have reported that females used the footbridge more than males [6, 24]. Yet, in other studies like this study, the gender differences was not significant [15, 22].

Although there were no differences between age group and education with bridge users in this study, many studies mention these factors as the influencing factors for these behaviors [6, 21, 24]. These differences seemed to be due to a balance between the perceived value of crossing and the perceived risk.

Furthermore in this study, pedestrians who had driving license used pedestrians bridges less. This finding is contrary to the finding of the previous study in China [6, 24]. It is expected that individuals with driving licenses become more familiar with the rules and follow them more. But the impact of environmental factors seems to be greater.

This study showed that morning and evening peak hours had higher users than off-peak hours such as other studies [15, 17]. It revealed that the usage rate of the pedestrian bridge is linearly proportionate with the traffic volume.

The most reasons for bridge use were safety and security which were also mentioned in other studies [6, 13, 15, 17-20, 24]. The main purpose of the implementation of pedestrian bridges is to maintain the health and safety of pedestrians. What is important is the factors influencing the non-use of bridges despite the perceived risk of crossing the streets illegally. In this study, seeing bridge use as time-saving, laziness, the inadequacy of the bridge, slipping stairs and steep of the bridge were the most effective factors on non-use of bridges. Previous studies have also emphasized the standardization of bridges. The fact that bridges have to be placed in the right place with the right conditions is very effective in their use by pedestrians [6, 12, 16, 17, 19, 20, 23-27]. The best motivation to encourage pedestrians using the bridge is to remove these barriers.

Unfortunately, the characteristics of bridges were not viewed according to the height, width, length, number of steps, the width of streets under them, number of lanes and existence of fences. It is highly recommended that these points be considered in future studies. 

The pedestrian bridge should be designed for vulnerable groups such as the elderly, children and the disabled. Lack of attention to the comfort and safety of pedestrians on these bridges is effective on their non-use. Therefore, in order to increase the use of the bridge, it is necessary to resolve all the barriers to its use.

## Declaration

### Ethics approval and consent to participate:

This study was approved by Research Ethics Committee of Guilan University of Medical Sciences (code:IR.GUMS.REC.1396.3694). Verbal consent was received from all the participants before enrollment.

### Consent for publication:

None declared.

### Conflicts of interest:

There are no conflicts of interest.

### Funding:

None declared. 

### Author’s Contribution:

Iraj Barge-Gol has taken a significant part in Conception or design of the work, Data analysis and interpretation, Drafting the article, Critical revision of the article.

Final approval of the version to be published. Leila KouchakinejadEramsadati has participated in Conception or design of the work, Data collection and Drafting the article. Payam Abedian and Helya Jafari-Shakib were involved in Conception or design of the work, Data collection and Drafting the article.

### Acknowledgment:

The authors would like to offer their special thanks to Guilan Road Trauma Research Center for their financial support and Ms. Fatemeh Javadi for editing the language of the manuscript. 

## References

[1] World Health Organization Road traffic injuries.

[2] GBD 2015 Mortality and Causes of Death Collaborators (2016). Global regional, and national life expectancy, all-cause mortality, and cause-specific mortality for 249 causes of death, 1980-2015: a systematic analysis for the Global Burden of Disease Study 2015. Lancet.

[3] Mathers CD, Loncar D (2006). Projections of global mortality and burden of disease from 2002 to 2030. PLoS Med.

[4] Bazargan-Hejazi S, Ahmadi A, Shirazi A, Ainy E, Djalalinia S, Fereshtehnejad SM (2018). The Burden of Road Traffic Injuries in Iran and 15 Surrounding Countries: 1990-2016. Arch Iran Med.

[5] World Bank group Global Road Safety Facility. Road Safety Country Profile, Iran.

[6] Holland C, Hill R (2007). The effect of age, gender and driver status on pedestrians’ intentions to cross the road in risky situations. Accid Anal Prev.

[7] Organization WH (2013). Pedestrian safety: a road safety manual for decision-makers and practitioners.

[8] Hasani J, Hashemi Nazari SS, Khorshidi A, Shojaei A (2016). Factors related to pedestrians mortality following road traffic accidents in Tehran and Alborz Provinces, Iran. International Journal of Epidemiologic Research.

[9] Daneshpour SA, Mahmoudi M, Abbasi B (2014). Evaluation of Pedestrian Level of Service for Tehran Crosswalks; Case Study of Nabovat Square in East of Tehran. InCIEC 2013: Springer;.

[10] Yadollahi M, Gholamzadeh S (2019). Five-Year Forecasting Deaths Caused by Traffic Accidents in Fars Province of Iran. Bull Emerg Trauma.

[11] Tabari P, Shabanikiya H, Bagheri N, Bergquist R, Hashtarkhani S, Kiani F (2020). Paediatric, pedestrian road traffic injuries in the city of Mashhad in north-eastern Iran 2015-2019: a data note. BMC Res Notes.

[12] Abojaradeh M (2013). Evaluation of pedestrian bridges and pedestrian safety in Jordan. Civil and Environmental Research.

[13] Malik IA, Alwi SKK, Gul N (2017). A survey to understand people perception of pedestrian bridges usage on Shahrah-e-Faisal road, Karachi-Pakistan. New Horizons.

[14] Manjanja RA (2013). Non-usage of pedestrian footbridges in Kenya: The case of Uthiru Pedestrian footbridge on Waiyaki way: University of Nairobi.

[15] Arsandrie Y, Prakoso A (2020). The need of pedestrian footbridge to promote sustainable living; case study: footbridge Jln A Yani Pabelan, Kartasura. Civil Engineering and Architecture.

[16] Ramadani HN, Rahmani H, Gazali A, editors Study of efficiency pedestrian bridge crossing in the road of Pangerang Antasari, Banjarmasin. MATEC Web of Conferences; 2018: EDP Sciences.

[17] Oviedo-Trespalacios O, Scott-Parker B (2017). Footbridge usage in high-traffic flow highways: The intersection of safety and security in pedestrian decision-making​. Transportation research part F: traffic psychology and behaviour..

[18] Hełdak M, Kurt Konakoglu SS, Kurdoglu BC, Goksal H, Przybyła B, Kazak JK (2021). The Role and Importance of a Footbridge Suspended over a Highway in the Opinion of Its Users—Trabzon (Turkey). Land.

[19] Hasan R, Napiah M (2017). Utilization of footbridges: Influential factors and improvement proposals. Advances in Transportation Studies..

[20] Räsänen M, Lajunen T, Alticafarbay F, Aydin C (2007). Pedestrian self-reports of factors influencing the use of pedestrian bridges. Accid Anal Prev.

[21] Hidalgo-Solórzano E, Campuzano-Rincón J, Rodríguez-Hernández JM, Chias-Becerril L, Reséndiz-López H, Sánchez-Restrepo H (2010). Motivos de uso y no uso de puentes peatonales en la Ciudad de México La perspectiva de los peatones [Use and non-use of pedestrian bridges in Mexico City The pedestrian perspective]. Salud Publica Mex.

[22] Truong LT, Nguyen HTT, Nguyen HD, Vu HV (2019). Pedestrian overpass use and its relationships with digital and social distractions, and overpass characteristics. Accid Anal Prev..

[23] Mohtasham-Amiri Z, Dastgiri S, Davoudi-Kiakalyeh A, Imani A, Mollarahimi K (2016). An Epidemiological Study of Road Traffic Accidents in Guilan Province, Northern Iran in 2012. Bull Emerg Trauma.

[24] Wu Y, Lu J, Chen H, Wu L (2014). Identification of contributing factors to pedestrian overpass selection. Journal of traffic and transportation engineering (English edition).

[25] Zare Zareharofteh F, Eslami M (2021). Pedestrians’ Outstanding Beliefs Regarding Bridge Use–A Directed Content Analysis. Health Education and Health Promotion.

[26] Rai A, Tiwari SR, editors (2019). Implication of Land Use and Street Characteristic on Overhead Pedestrian Bridge Usage. Proceedings of IOE Graduate Conference;.

[27] Rahmawati Y, Firzal Y (2020). Study of User’s Response on the Pedestrian Bridge in Pekanbaru City. Journal of Physics: Conference Series.

